# Percutaneous Tricuspid Valve Repair

**DOI:** 10.31083/j.rcm2307220

**Published:** 2022-06-24

**Authors:** Antonio Mangieri, Alessandro Sticchi, Aisha Gohar, Damiano Regazzoli, Fabio Fazzari, Daniela Pini, Marta Pellegrino, Beniamino Pagliaro, Ferdinando Loiacono, Mauro Chiarito, Bernhard Reimers, Fabien Praz, Azeem Latib, Antonio Colombo

**Affiliations:** ^1^Department of Biomedical Sciences, Humanitas University, Pieve Emanuele-Milan, Italy and IRCCS Humanitas Research Hospital, 20089 Rozzano-Milan, Italy; ^2^Department of Cardiology, Bern University Hospital, University of Bern, 3012 Bern, Switzerland; ^3^Department of Cardiology, Montefiore Medical Center, Bronx, NY 10466, USA

**Keywords:** tricuspid valve, tricuspid regurgitation, percutaneous tricuspid valve repair, heart failure, right ventricle, right heart failure

## Abstract

Tricuspid regurgitation (TR) negatively affects patient outcomes. Surgical tricuspid valve repair/replacement carries a high operative risk and is not a viable option for many high-risk patients. Percutaneous approaches provide an attractive alternative solution for such patients since they represent a valid alternative to open heart surgery without the significant risks carried by surgery. A number of percutaneous devices are currently under clinical development. This review will discuss about the latest development in the field of percutaneous tricuspid valve repair with possible future developments.

## 1. Introduction

Tricuspid regurgitation (TR) has long been neglected and considered a benign form of valvular heart disease with no clinically relevant sequalae if managed conservatively. However, in recent years corroborating evidence suggests that without intervention, TR can negatively affect prognosis. Historically, TR has been treated surgically using various plasty techniques targeting annular dilatation, the most common anatomical mechanism seen with TR. Patients undergoing tricuspid plasty have a relatively good outcome when the repair is performed concomitantly with left-sided valvular repair/replacement; on the contrary, patients undergoing isolated tricuspid valve repair/replacement have a poor prognosis with an in-hospital mortality of 8.8%, a rate which has not improved over time despite an increase in volume of procedures performed [1]. Percutaneous interventions are therefore a more attractive alternative solution as whilst involving a similar technique as surgical repair, the associated improvement of symptoms and prognosis comes without the high rate of periprocedural complications [2, 3, 4]. A recent pooled analysis of 771 patients who underwent transcatheter tricuspid valve intervention showed a significant improvement of functional status at 6 month follow-up [5]. This review focuses both on the options currently available for percutaneous tricuspid valve repair and on future directions.

## 2. Anatomical Basis of Tricuspid Valve Repair

### 2.1 The Leaflets 

**Fig. 1.**
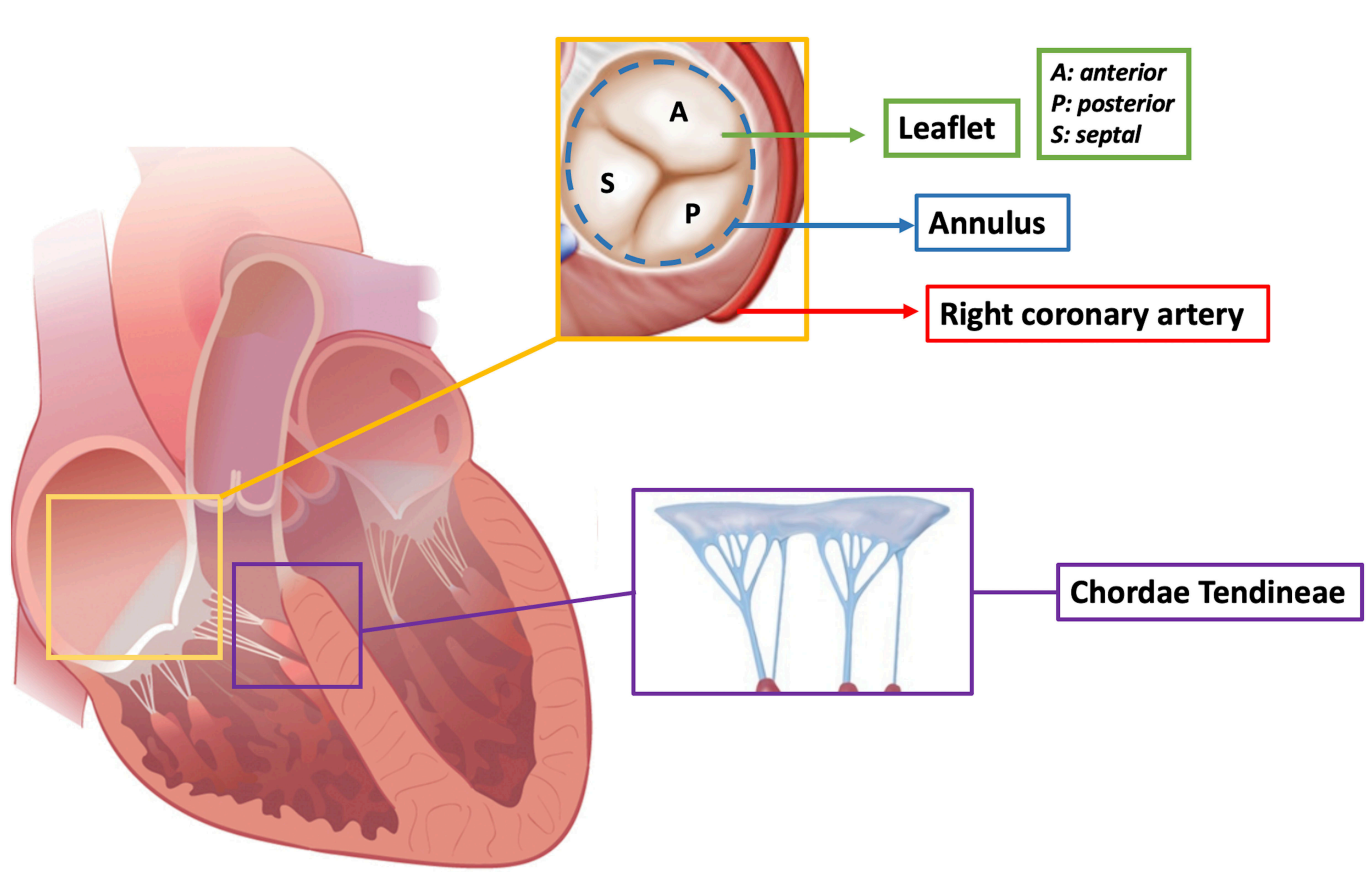
**Anatomy of the tricuspid valve**.

The tricuspid valve is a complex anatomical entity characterized by a well-defined structure with huge anatomical variability especially in the composition of the leaflets (Fig. 1). According to newly standardized recently proposed nomenclature, the tricuspid valve has three well defined leaflets in only ∼54% of patients and four functional leaflets are detectable in ∼39% of patients. The most common leaflet composition is the two posterior leaflet morphology. This huge anatomical variability in leaflet composition has important implications in valve repair as the four leaflet configuration can be associated with an increased risk of residual TR post procedure. The surface of the leaflets is divided into three zones: (1) the rough zone, onto which most of the chordae tendineae are inserted, (2) the basal zone, and (3) the clear zone, which lies between the rough and basal zones.

### 2.2 Chordae

There are five types of chordae, classified according to their morphology and site of contact with the leaflets: fan-shaped, rough zone, basal, free edge and deep chordae [6]. The chordal apparatus is redundant hence why TR secondary to flail leaflets is rare. Moreover, due to the presence of multiple true and false chordae in the right ventricle, a trans-apical approach for percutaneous tricuspid valve repair is challenging. Notably, leaflet-based technologies which aim to reduce TR through leaflet grasping should take into account the risk of device entrapment in the chordal apparatus. Interestingly, a sub-annular device has been developed (Mistral device-Mitralix, Yok’neam, Israel); its mechanism of action consists of grasping the chordae together and reshaping the valve, hence favoring the reduction of TR [7].

### 2.3 Annulus

In contrast to the mitral valve, the tricuspid apparatus has a virtual annulus which lies at the level of the atrio-ventricular groove. It is largely composed of fibroadipose tissue, which spans almost the entire circumference of the valvular orifice, excluding the area where the hinge of the septal leaflet is connected to the membranous ventricular septum.

The normal tricuspid annulus is elliptical with a larger mediolateral diameter; its shape mimics the saddle shaped mitral annulus with a higher antero-septal portion. In the presence of functional TR, the tricuspid annulus becomes more planar, dilating primarily in the septal-lateral direction, producing a more circular shape compared to the native anatomy [8]. The anatomical features of the tricuspid annulus have direct implications on the development of trans-catheter devices:

-The right coronary artery runs along the atrio-ventricular groove for the first portion of its course. Annuloplasty systems, which need to be anchored at the hinge point of the tricuspid valve, can distort the surrounding tissues with consequential spasm of the right coronary artery [9]. Close anatomical continuity between the hinge point and the right coronary artery is a contraindication to the Cardioband (Edwards Lifescience, Irvine, CA, USA) implant. Real-word experience using the Cardioband system resulted in a right coronary artery stent implantation rate of 11.7% [10].

-The bundle of His, which penetrates the central fibrous body and runs beneath the membranous septum 3–5 mm from the antero-septal commissure, can be damaged during transcatheter tricuspid valve implantation resulting in permanent pacemaker implantation requirement. The first-in-man experience with the transfemoral Evoque valve system (Edwards Lifescience, Irvine, CA, USA) reported a rate of 8% of permanent pacemaker implantation [11]. In the case of incomplete percutaneous annuloplasty system implantation the rate of permanent pacemaker implantation is 3% [10].

-The tricuspid valve annulus is fragile and in close proximity to the free wall of the right ventricle. Damage as a result of the implantation of anchors and/or devices can occur not infrequently resulting in cardiac tamponade.

## 3. Pros and Cons of Tricuspid Valve Repair 

**Fig. 2.**
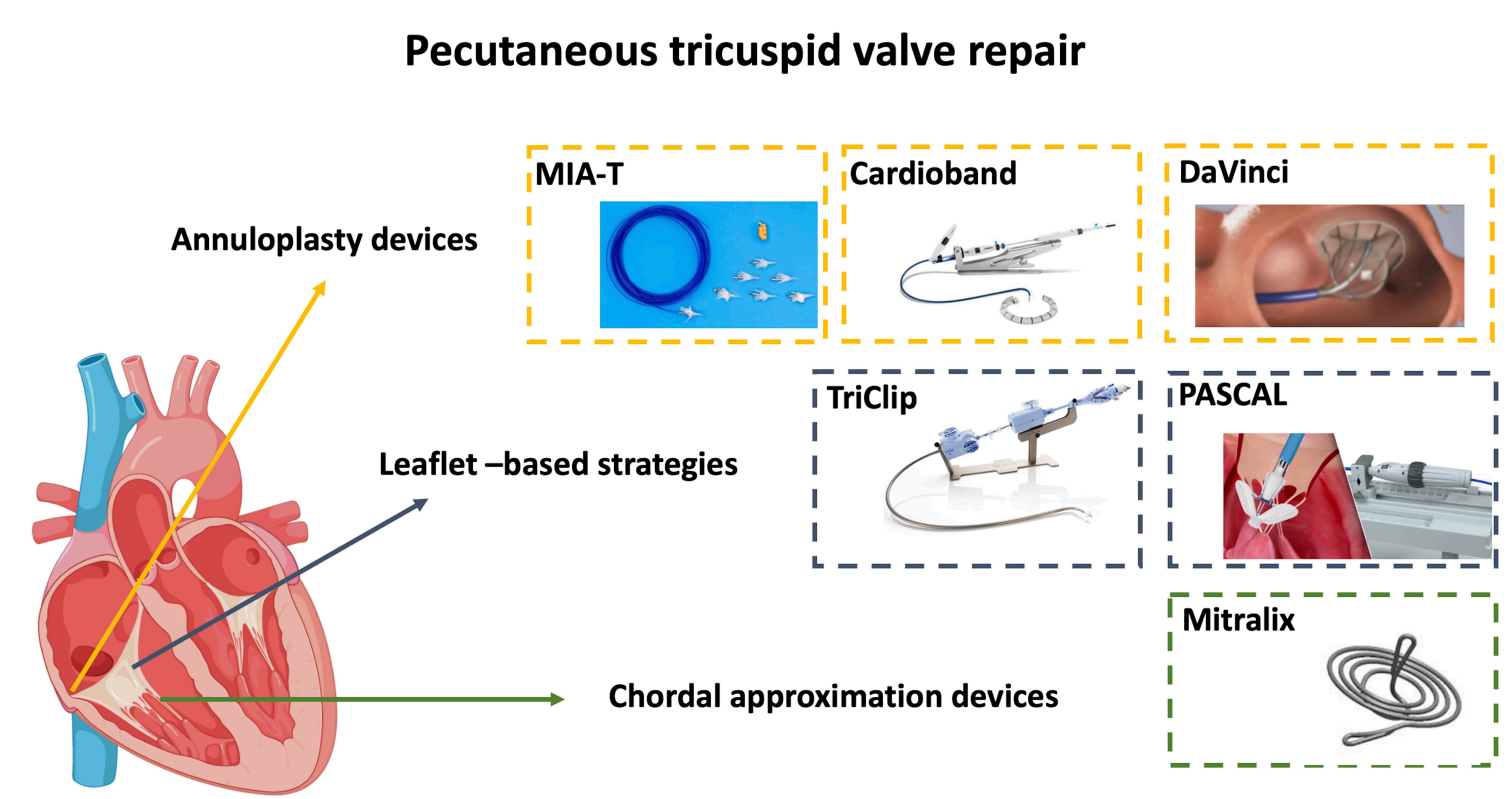
**Tricuspid valve repair systems currently under clinical development**.

Surgical tricuspid valve repair is preferred over replacement for many reasons, in particularly due to good long term follow-up results when repair was performed using rigid prosthetic rings [12]. As the percutaneous field progresses, innovative percutaneous reparative solutions are being developed (Fig. 2). However, cardiologists are seeing a number of pros and cons in the transcatheter repair technique of the tricuspid valve.

### 3.1 Pros

-Preservation of native anatomy: whilst treating TR it is possible to preserve the native anatomy. For example with percutaneous annuloplasty, anatomy of the leaflets is preserved allowing additional interventions using leaflet-based plasty systems to be performed.

-No need for strict antithrombotic therapy: contemporary percutaneous repair devices do not require any particular antithrombotic therapy therefore patients treated with annuloplasty and leaflet plasty systems can be managed without anticoagulation. To date, no cases of thrombosis have been reported following transcatheter edge-to-edge repair (TEER) or annuloplasty. Conversely, with transcatheter tricuspid valve replacement, strict antithrombotic regimes are needed to avoid the risk of thrombosis.

-Versatility of the treatment: with TEER, in the case of leaflet-based therapy, patients can be screened with only transesophageal echocardiography (TOE) without the need for computed tomography (CT) imaging. Annuloplasties however usually require detailed CT scan planning. This is particularly advantageous for patients with severe chronic kidney disease who would otherwise be exposed to nephrotoxic contrast medium pre-procedurally in addition to the procedural contrast use.

-No preclusion for future intervention: in case of failure of percutaneous annuloplasty, possible future intervention with leaflet based plasty remains possible.

### 3.2 Cons

-Risk of suboptimal results: in patients with severe TR, intervention using percutaneous repair systems can frequently result in suboptimal results. This is related to the complex anatomy of the tricuspid valve with multiple concomitant mechanisms that contribute to the regurgitation. In particular, both atrial and ventricular TR are associated with a degree of annular dilatation which can be treated with annuloplasty but is rarely fully reversed with TEER [13]. Moreover, a posteriorly located jet and a large coaptation gap independently predict isolated TEER failure [14], indicating that complex anatomies with advanced remodeling affect treatment response.

-Requirement for adequate intraprocedural imaging: percutaneous tricuspid valve repair requires meticulous intraprocedural visualization of the valve anatomy. TOE is essential for TEER to identify the grasping area and to guide the delivery system across the valve to below the valvular plane. Good visualization of the arms is essential to be able to orientate the device and to obtain solid grasping avoiding single leaflet detachment and loss of leaflet insertion. To overcome these limitations, 2 three/four-dimensional intracardiac echocardiography (3D/4D ICE) systems have been developed — the VeriSight Pro ICE (Philips- Amsterdam, Netherlands) and the NuVision Ultrasound Catheter (Biosense Webster, Irvine, CA, USA) offering real-time, detailed anatomy of the tricuspid leaflets and of the surrounding structures. The use of 3/4D ICE to guide TEER has the potential benefit of reducing the rate of detachment whilst allowing faster and more efficient percutaneous annuloplasties to be performed.

## 4. Leaflet Based Technologies 

### 4.1 TriClip 

**Fig. 3.**
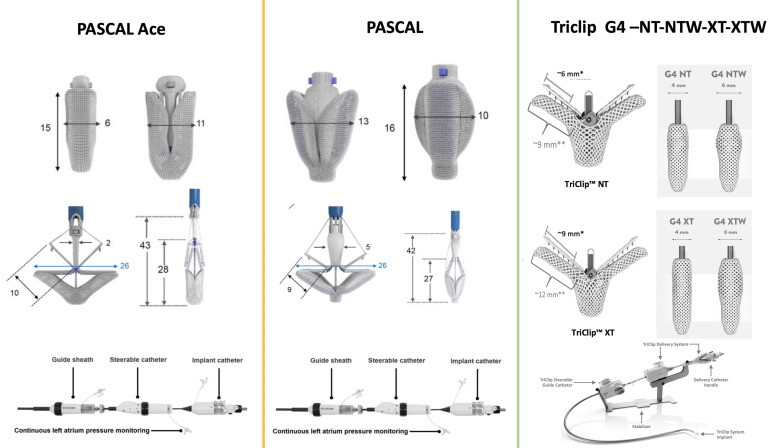
**Available transcatheter tricuspid valve repair systems for tricuspid regurgitation**.

The TriClip (Abbott, Santa Clara, CA, USA) system is built upon a proven clip-based platform that is uniquely designed for the tricuspid valve. Similarly to the MitraClip, the TriClip system consists of a steerable guiding catheter and a clip delivery system which have been modified to navigate the right heart. In contrast to the MitraClip system, the steerable guiding catheter has two additional knobs for facilitating the steering maneuvers while the steerable sleeve only has one knob for deflecting the distal tip over a shorter radius. As a result of these changes, TriClip is able to be rotated and directed towards the tricuspid valvular plane. Different generations of the device have been developed with longer and wider arms allowing for more efficient grasping (Fig. 3).

Following initial international experience of 64 consecutive cases of TEER using the MitraClip in the tricuspid position, subsequent studies have confirmed the efficacy of TriClip in reducing the grade of TR in selected patients. The Transcatheter Clip Repair System in Patients With Moderate or Greater TR (TRILUMINATE) trial enrolled 85 patients with at least moderate TR treated with the TriClip system. At 6 months follow-up, the rate of less than moderate TR was 57% and a reduction of at least one grade of regurgitation was obtained in 71% of the patients. Patients experienced an improvement in their functional class at follow-up with a low rate of periprocedural and post procedural complications. Of note, single leaflet detachment was observed in 7% of patients [4].

Real world experience with the use of XTR TriClip, including 50 patients with at least moderate TR, showed a technical success rate of 100% with a mean average of two clips implanted per patient; 88% of the clips were implanted in the antero-septal commissure. At 30 days follow-up, less than severe TR was achieved in 43% of patients; interestingly, a sub optimal result was more frequently achieved in patients with a large coaptation gap (>10 mm). Single leaflet attachment occurred in five (7%) of 72 patients [15]. The initial experience using TriClip in a real-world setting was recently presented as part of the Observational Real-world Study Evaluating Severe Tricuspid Regurgitation Patients Treated With the Abbott TriClip™ Device (bRIGHT) registry. 200 patients treated with the TriClip device were included (7% received a G4 TriClip). The authors reported that 98% of patients had successful implantation of at least one clip and 88% of patients had acute procedural success (defined as a reduction in TR of at least one grade). At 30-day follow-up, 66% of patients were left with moderate or less TR. Three patients required re-intervention or reoperation and 10 had single-leaflet device attachment [16]. In the near future, efficacy of TEER with TriClip will be evaluated in the TRILUMINATE Pivotal Trial (NCT03904147) comparing invasive percutaneous treatment with medical therapy.

### 4.2 PASCAL

The PASCAL (Edwards Lifescience, Irvine, CA, USA) TEER system enables percutaneous leaflet repair of the regurgitant tricuspid valve by means of tissue approximation. Its unique features include 2 spring-loaded paddles of 25 mm in width, 2 clasps of 10-mm in length and a 10 mm central spacer. The PASCAL Ace is a second generation device with a narrower central spacer and profile, which helps leaflet grasping of the tricuspid valve (Fig. 3). The first experience of TR with the PASCAL device included a retrospective analysis of 30 patients. All patients presented with at least severe TR which, in most cases was functional in etiology (83%). Moderate or less than moderate TR was achieved in 86% of patients at 12 months. Single leaflet detachment was observed in two patients [17]. In another case series including 28 patients treated with PASCAL for significant TR, a satisfactory reduction in TR with less than moderate residual regurgitation was obtained in 85% of cases. Two cases of detachment occurred and were managed conservatively [18].

Treatment of TR with the PASCAL Ace system (Edwards Lifescience, Irvine, CA, USA) was described in 16 patients with severe or more than severe TR. Eleven procedures (69%) resulted in successful reduction in TR. In four patients, PASCAL Ace implantation was unsuccessful because of poor imaging quality and a hostile anatomical environment; one patient did not achieve TR reduction despite successful TEER [19].

In a propensity matched analysis, PASCAL had similar outcomes compared to the MitraClip-XTR group with moderate or less than moderate TR observed in 11 (50%) patients in the PASCAL group and 15 (68%) patients in the MitraClip-XTR group (*p *= 0.56) [20].

## 5. Chordal Approximation Devices

### 5.1 Mitralix 

The Mistral technology (Mitralix, Yok’neam, Israel) has been developed as an innovative percutaneous treatment for TR. The device consists of a delivery system and a spiral-shaped single nitinol wire 0.475 mm in diameter. The technology is based on sinching of the sub annular apparatus through the grasping of tricuspid chordae, creating a “flower bouquet” that allows reshaping with consequent reduction in TR. A study of the first-in-man experience reported the 30-day outcome of six patients. The procedures were uneventful and demonstrated a reduction in the effective regurgitant area from a median of 0.52 ${\mathrm{cm}}^{2}$ at baseline to 0.15 ${\mathrm{cm}}^{2}$ at 30 days follow-up (*p *< 0.01); concordantly, vena contracta width was reduced from 0.95 cm to 0.62 cm (*p *< 0.05) and regurgitant volume decreased from 49.4 mL/beat to 19.7 mL/beat (*p *< 0.01). The MATTERS (Mistral Percutaneous Tricuspid Valve Repair FIM Study) II early feasibility trial is currently ongoing in Europe (NCT04073979). The trial aims to demonstrate acute device safety and technical performance along with longer follow-up device safety and efficacy evaluation. The device performance will be defined according to the capability of implanting the device correctly with the grasping of at least two leaflets.

### 5.2 Annuloplasties 

Transcatheter annuloplasty is one of the most effective repair techniques derived from surgery, and is associated with improved survival and lower re-intervention rates [21].

Anatomy plays an important role in the feasibility of this technique. According to the last dedicated experts’ consensus, this technique is advocated in cases of mild-to-moderate annular dilatation as the primary mechanism of TR, mild-to-moderate tethering, central jet location, and sufficient landing zone for anchoring after assessment of the right coronary artery. The presence of a cardiac implantable electronic device (CIED) lead is not an absolute contraindication if it does not impinge on the leaflets [22].

In the recent past, incomplete percutaneous annuloplasty systems (Trialign, Tricinch) have demonstrated doubtful results with scarce evidence in terms of TR reduction and valve remodelling.

Other complete annuloplasty systems (Millipede, Boston Scientific) have been abandoned due to difficulties in clinical development. Currently there are new technologies that are in the clinical and pre clinical development phases.

### 5.3 Cardioband 

The Cardioband™ direct annuloplasty system (Edwards Life-sciences, Irvine, CA, USA) is the most studied device with long-term results. It received the CE mark for the treatment of patients with severe symptomatic secondary TR in 2018 after the TRI-REPAIR (TrIcuspid Regurgitation RePAIr With CaRdioband Transcatheter System study) [23]. The procedure consists of the implantation of an adjustable band on the valve annulus using a number of screws as anchors. The annular device is positioned via a transfemoral delivery system, with a shrinkable steerable sheath (24 Fr) band in order to achieve the annuloplasty degree required for treating the regurgitation under echocardiographic guidance.

The TRI-REPAIR study is a single-arm, multicenter, prospective study which evaluated the safety and efficacy of the Cardioband system in 30 patients. It showed a sustained reduction in TR to moderate or less at 6 months in 73% of the population and in 72% of the population at 2 years [23].

Furthermore, the recently published two-year follow up data highlighted a significant reduction in septolateral annular diameter of 16% (*p* = 0.006) compared to 9% in the first report, and a stable improvement in New York Heart Association (NYHA) functional class with more than 80% of cases in class I-II (*p *= 0.002).

The trend at two years was confirmed in the post-market TriBAND study showing a reduction in the septolateral diameter of 20%, reaching 69% of patients with TR ≤2 at 30 days. Moreover, the population in the TRI-REPAIR and TriBAND represented a more severe disease phenotype, evidenced by larger EROA at baseline in comparison with TRILUMINATE (0.79 ± 0.51 ${\mathrm{mm}}^{2}$, 0.76 ± 0.48 ${\mathrm{mm}}^{2}$ and 0.65 ± 0.03 ${\mathrm{mm}}^{2}$, respectively) [4, 23, 24].

Finally, the first real-world experience confirmed the feasibility of direct annuloplasty with a safe learning curve using Cardioband to treat TR. Sixty consecutive patients at four German centers were included retrospectively showing a less-than-severe TR at discharge in 60.3% of patients. At follow-up, 81.3% of patients were in NYHA class I or II [10]. One of the most feared device-related complications concerns right coronary artery perforation which occurred in nine patients (15%) [25]. Partial vessel deformation on the other hand is usually transient and rarely requires stent implantation. Patient selection and careful procedural planning remain fundamental to this procedure, but additional device and technical improvements are guaranteed. The Edwards Cardioband Tricuspid Valve Reconstruction System Early Feasibility Study (NCT03382457) will expand on the knowledge regarding the use of the Cardioband system in a cohort of 55 patients.

### 5.4 MIA-T

The MIA-T system is a sutureless transcatheter annuloplasty system consisting of low-mass, polymeric, self-tensioning PolyCor anchors and a thermoplastic elastomer (MyoLast) which tensions the anchors inducing annular plication. The system uses a dedicated 12 Fr delivery catheter and has been investigated by the Study of Transcatheter Tricuspid Annular Repair trial (STTAR; ClinicalTrials.gov Identifier: NCT03692598) using both the surgical and transcatheter approaches. The system achieves a bicuspidization of the valve by obliterating the posterior leaflet. The trial is still recruiting and no data has been published, however the company recently submitted the technical documentation for CE mark approval [26].

### 5.5 DaVingi 

The DaVingi™ TR system (Cardiac Implants, Tarrytown, New York) is a two-step procedure using a novel trans jugular approach allowing complete annuloplasty. During the first procedure, the device is deployed using a dedicated multi-arm scaffold which allows the simultaneous implantation of anchors onto the atrial aspect of the tricuspid valve. Predictable annular physiological constriction using an adjustment tool takes place in the second stage (90 days) after a period of tissue healing [26]. The first clinical trial of 15 patients is currently undergoing recruitment (NCT03700918).

### 5.6 Long Term Outcomes of Transcatheter Tricuspid Valve Repair 

Currently, little clinical data are available demonstrating the safety and efficacy of transcatheter tricuspid valve repair. The one year follow-up of the TRILUMINATE trial demonstrated the sustainability of the TEER in a selected cohort of patients. At 1 year, TR was reduced to moderate or less in 71% of subjects compared with 8% at baseline (*p *< 0.0001). Patients experienced significant clinical improvement in NYHA functional class I/II, 6-minute walk test (272.3 ± 15.6 to 303.2 ± 15.6 meters, *p* = 0.0023) and Kansas City Cardiomyopathy Questionnaire (KCCQ) score (improvement of 20 ± 2.61 points, *p *< 0.0001) [27]. Similar results were observed for the Edwards PASCAL Transcatheter Valve Repair System Pivotal Clinical (CLASP TR) Trial (NCT04097145): the one year outcome demonstrated an improvement of at least two 2 grades of TR in 75% of patients and 86% of patients had less than moderate or moderate residual TR at one-year follow-up [28].

### 5.7 Criteria for Optimal Results in Transcatheter Tricuspid Valve Repair 

Optimal patient selection plays an important role in achieving good results using percutaneous treatment for tricuspid valve repair. In the case of TEER, the presence of a coaptation gap larger than 7.2 mm and a non-central/non antero-septal TR jet location are the strongest predictors of suboptimal TEER [14]. Recently a low leaflet-to-annulus index (calculated as anterior leaflet length plus septal leaflet length)/septolateral tricuspid annulus diameter) was shown to be associated with the risk of more than moderate residual TR after TEER [29]. Notably, the presence of a CIED lead across the tricuspid valve does not have a negative impact on procedural outcome [30]. In the case of percutaneous annuloplasty, an optimal result can be obtained in the absence of concomitant leaflet pathology and/or tethering of the leaflets.

## 6. Future Prospective 

Transcatheter intervention on the tricuspid valve is continuously growing and offers a wide range of solutions to patients suffering from TR. Unfortunately, the complexity of the tricuspid valve apparatus and sub-apparatus makes results from repair mostly unpredictable and far from perfect. This understandably leads one consider replacement over repair, but several questions remain unanswered with various aims needing to be met that go beyond achieving complete reduction of TR.

The complete abolishment of TR is not desirable in every patient and the degree of improvement required to be beneficial is determined by the right ventricle function. Several patients are too fragile to receive anticoagulation therapy needed to reduce the risk of thrombosis as a result of the low-pressure right heart circulation. In addition, the pathological degeneration of TR results in annuli dimensions that are too large and unsuitable for current replacement devices. These concerns mean that the treatment for TR is still an open field in need of technological innovation and further studies.

## 7. Conclusions

Tricuspid valve intervention continues to be a field of uncertainty. The challenge of meeting short term safety and efficacy goals, like long term durability, only scratches the surface of this complex scenario. New percutaneous devices have been proven to show safety and efficacy in the first-in-human experiences. Nether the less new reparative techniques are still some way from surgical techniques in terms of efficacy. Further studies are required with long-term follow up before they can be established into routine clinical practice.

## Abbreviations

TR, tricuspid regurgitation; TEER, transcatheter edge-to-edge repair; CT, computed tomography; TOE, transoesofageal echocardiography; CIED, implantable electronic device; NYHA, New York Heart Association.
